# The effects of relaxation techniques following acute, high intensity football training on parasympathetic reactivation

**DOI:** 10.3389/fspor.2023.1267631

**Published:** 2023-11-28

**Authors:** Eric Renaghan, Michael Joseph Wishon, Harrison L. Wittels, Luis A. Feigenbaum, Kyle Bellamy, Michael Hatfield, Joe Girardi, Stephen Lee, Samantha M. McDonald, S. Howard Wittels

**Affiliations:** ^1^ Department of Athletics, Sport Sciences, University of Miami, Miami, FL, United States; ^2^ Tiger Tech Solutions, Inc., Miami, FL, United States; ^3^ Department of Physical Therapy, Miller School of Medicine, University of Miami, Miami, FL, United States; ^4^ Department of Athletics, Nutrition, University of Miami, Miami, FL, United States; ^5^ Marine Special Operations Command (MARSOC), Camp Lejeune, NC, United States; ^6^ United States Army Research Laboratory, Adelphi, MD, United States; ^7^ School of Kinesiology and Recreation, Illinois State University, Normal, IL, United States; ^8^ Department of Anesthesiology, Mount Sinai Medical Center, Miami Beach, FL, United States; ^9^ Department of Anesthesiology, Wertheim School of Medicine, Florida International University, Miami, FL, United States; ^10^ Miami Beach Anesthesiology Associates, Miami Beach, FL, United States

**Keywords:** parasympathetic reactivation, autonomic nervous system, elite athletes, exercise training, American football, collegiate

## Abstract

**Background:**

Evidence shows relaxation techniques reactivate the parasympathetic nervous system (PNS) following physiological stressors such as exercise. As such, these techniques may be useful following exercise training of high intensity sports, like collegiate football.

**Purpose:**

To evaluate the impact of mindfulness and rest activities on PNS reactivation following training sessions, in a sample of Division-I collegiate, male football athletes.

**Methods:**

This study employed a cross-sectional, pre-post experimental design among 38 football athletes. Following three training sessions, each separated by one week, athletes were exposed to three groups: mindfulness, rest, and no-intervention. Athletes in the mindfulness group laid supine in a darkened room, while performing 15 min of guided breathing and body scans. The rest group remained seated in a lighted room, performing 15 min of restful activities (e.g., talking). The no-intervention group was instructed to perform usual post-training activities (e.g., showering). Heart rate (HR), respiration rate (RR) and two HR variability (HRV) indices were measured via an armband monitor (Warfighter Monitor, Tiger Tech Solutions, Inc, Miami, FL) equipped with electrocardiographic and photoplethysmography capabilities. HRV indices included standard deviation of the N-N intervals (SDNN) and root mean square of successive RR interval differences (rMSSD). Within and between-group differences were determined via analysis of variance (ANOVA) and corrected for multiple comparisons familywise error.

**Results:**

Statistically significant reductions in HR and RR were observed across all groups: −81.6, −66.4, −40.9 bpm and −31.7, −26.9, and −19.0 breaths⋅min^−1^, respectively. The mindfulness and rest groups exhibited a larger within-group reduction in HR and RR compared to the no-intervention group, *p* < 0.0000. Additionally, the mindfulness group showed a larger reduction in HR and RR compared to the rest group, *p* < 0.05. Post-intervention HR and RRs were significantly lower in the mindfulness group relative to the no-intervention group (77.0 vs. 120.1 bpm, respectively). Similar results were observed for RR (15.0 vs. 23.6 breaths⋅min^−1^, respectively) and HRV indices (SDNN: 46.9 vs. 33.1 ms and rMSSD: 17.9 vs. 13.8 ms, respectively) Athletes in the rest group showed significantly lower post-intervention HR (−30.2 bpm, 89.9 vs. 120.1 bpm, respectively), RR (−4.3 breaths⋅min^−1^, 19.3 vs. 23.6 breaths⋅min^−1^, respectively) and significantly higher HRV (SDNN: 42.9 vs. 33.1 ms and rMSSD: 16.7 vs. 13.8 ms, respectively) compared to their no-intervention counterparts.

**Conclusions:**

Our findings suggest that athletes engaging in either 15-minute guided mindfulness or rest activities (e.g., sitting) post training, may facilitate PNS reactivation. Implementing these strategies may accelerate recovery, improving performance. Longitudinal, randomized controlled trials among diverse sports are encouraged.

## Introduction

Exercise training activates the sympathetic nervous system (SNS) in direct proportion to intensity and duration (1). The SNS facilitates exercise performance through physiological responses like increases in skeletal muscle blood flow, blood pressure, cardiac output, heart rate, stroke volume, and respiration (2, 3). Post exercise, the SNS continues its dominance, supporting physiological recovery processes like replenishing myoglobin O_2_ stores, resynthesizing phosphocreatine, degrading hormones, increased glyconeogenesis from lactate accumulation, and removal of CO_2_ (4, 5). Eventually, the parasympathetic nervous system (PNS) reactivates, regaining homeostatic control (6). It is, in this state, an athlete, when provided sufficient time, may fully recover, progressively adapt, and subsequently, enhance their sport performance (7). Delays in reactivating the PNS, however, may prolong recovery as the sustained catabolic state inhibits processes such as full repletion of energy stores and repair of musculoskeletal damage (8–11). Thus, athletes may benefit from utilizing methods that reactivate the PNS immediately following training sessions, especially sessions of high volume and/or include high intensity exercise.

Substantial evidence shows that relaxation techniques such as mindfulness, progressive relaxation, autogenic training, and deep breathing positively influence the PNS (12, 13). Individuals implementing these techniques following various physiological stressors elicited reductions in heart rate (HR), respiration rates (RR), and increases in HR variability (HRV), indicating PNS reactivation (13). HR, RR, and HRV are indicators of the interplay between the PNS and SNS (i.e., sympathovagal balance). During parasympathetic dominance, acetylcholine released from cardiac nerve fibers slows the sinoatrial (SA) node, reducing HR. The controlled and slower inspirations and expirations observed in this state contribute in optimizing pulmonary gas exchange, venous return, and stroke volume (14, 15). Many studies previously demonstrated that deep and slowly controlled breathing, a primary component to relaxation techniques, regains PNS activation through these physiological mechanisms (16). Specifically, the activity of the efferent cardiac nerves fibers is at its peak throughout each prolonged expiration, progressively slowing HR and increasing HRV (12). During the lengthened inspiration, the expanding lung volume and intrathoracic pressure increases alveolar ventilation and perfusion, allowing for optimal loading and unloading of oxygen and carbon dioxide, respectfully, in addition enhanced cardiac blood flow (17, 18).

In the sports realm, current research shows that the anxiolytic effects of relaxation techniques are a powerful tool for elite athletes to use in reducing psychophysiological stressors that often precede competition (19). Athletes often experience high state anxiety, self-doubt, decreased confidence, and exhibit heightened activity of the SNS, all of which may negatively affect sport performance (19–21). Studies show that athletes implementing specific relaxation techniques like auditory and visual imagery, self-hypnosis, deep breathing, and progressive relaxation report reduced anxiety, positive self-image, increased self-efficacy and improved performance (22–24). Interestingly, however, previous studies showed that relaxation exercises following training sessions equivocally affected HR and HRV (25–27). Some factors potentially explaining these inconsistencies could be that studies relied on participants self-administering the intervention outside of training, resulting in self-reported compliance and its' overall effects on feelings of relaxation, stress, anxiety, and perceived performance (25, 27). As such, among athletes, the effects of relaxation techniques on physiological markers of PNS reactivation like HR, RR and HRV are less clear. Moreover, a dearth of literature exists on using relaxation techniques following acute bout of exercise training among athletes competing in high-intensity team sports like American football (28) and the effects on PNS reactivation.

Utilizing relaxation techniques following training sessions may greatly benefit collegiate football athletes for several reasons. Football athletes train with high frequency (5–6 sessions per week), longer duration (2–4 h) and at high intensities (29, 30). Consequently, these athletes may experience prolonged, elevated SNS activity, potentially delaying recovery (26). To our knowledge, no previous studies examined the influence of a brief relaxation intervention following an acute bout of training on PNS reactivation in collegiate, male football athletes. Therefore, the purpose of this study was to determine the effects of two, 15-minute relaxation exercises, mindfulness, or rest, on post-exercise HR, RR and HRV. We hypothesized that the athletes engaging in either mindfulness or rest techniques would elicit lower and larger reductions post-exercise HR and RR and higher increases in HRV compared to their no-intervention counterparts.

## Materials and methods

### Study design

This study employed a repeated-measures, non-randomized within-group experimental design. The participants were not randomized due to (1) the rigid structure of the training sessions requiring an efficient approach and (2) the within-group study design allowed for participants to serve as their own control reducing the individual variability for between-group comparisons. Thirty-eight male, collegiate football athletes were agreed to participate in each of the three interventions (mindfulness, rest, or no-intervention), following routine exercise training sessions. HR, RR and HRV were measured prior to and throughout both the training session and 15-minute intervention.

### Subjects

The study sample consisted of 38 collegiate football players recruited from one Division-I university located in the southeastern US. The prospective participants were recruited from a pre-selected group of athletes the coaches identified as “starters”, which were athletes that competed in nearly every regulation game and for most of its duration. On average, the total sample of athletes were 19.9 ± 1.4 years of age and ranged between 18.0 and 23.0 years. Twenty-nine percent of the athletes were classified as obese, according to body mass index values and most of the athletes were non-Hispanic black (68.4%). Importantly, no statistically significant differences in demographic characteristics were observed between the intervention groups (see Table 1). Prior to any measurements, the athletes were informed of the benefits and risks of the study and voluntarily consented to the study. All study protocols followed the ethical principles defined in the declaration of Helsinki and were approved by the University of Miami's Institutional Review Board (IRB #20191223).

**Table 1:** Demographic Characteristics of the Sample of Division-I Collegiate Football Athletes.

	Total (*n* = 38)	Mindfulness (*n* = 35)	Rest (*n* = 36)	No-intervention (*n* = 33)
	Mean (SD)	Mean (SD)	Mean (SD)	Mean (SD)
Age (years)	19.9 (1.4)	19.9 (1.4)	19.9 (1.4)	19.9 (1.4)
Anthropometrics				
Weight (kg)	105.7 (22.3)	106.0 (22.1)	105.6 (21.9)	102.8 (19.7)
Height (cm)	189.1 (7.0)	189.4 (6.9)	189.3 (7.1)	188.4 (7.0)
BMI (kg/m^2^)	29.3 (4.7)	29.4 (4.7)	29.3 (4.6)	28.8 (4.3)
% Obese	29.0	28.6	27.8	25.9
Race/Ethnicity (%)				
NH White	15.8	17.1	16.7	12.9
NH Black	68.4	65.7	66.7	67.7
NH Other	132.	14.3	13.9	16.1
Hispanic	2.6	2.9	2.8	3.2
Resting ANS Activity				
Pre-Training HR (bpm)		63.0 (7.2)	61.3 (6.3)	61.4 (8.1)
Pre-Training rMSSD (ms)		48.3 (0.4)	51.1 (0.6)	50.3 (0.7)
Pre-Training SDNN (ms)		80.7 (1.6)	84.2 (2.1)	82.7 (2.4)

NH,  non-Hispanic; SD, standard deviation; BMI, body mass index; ANS, autonomic nervous system; HR, heart rate; rMSSD, root mean square of the standard deviation of N-N intervals; SDNN, standard deviation of N-N intervals; RR, respiratory rate.

### Exercise training session

For the current study, one weekly exercise training session was selected per week across a 3-week period, occurring during their preseason summer football camp. Each session occurred on a Monday, separated by one week, lasted between 140 and 150 min and consisted of high intensity exercise. The process for selecting training sessions ensured the pre-intervention exposures were similar in both duration and intensity. All athletes were exposed to the same exercises including strength- & power-focused resistance exercises, short-distance sprint intervals, aerobic training, and agility training (31–33). The average post-exercise HR and RR prior to the interventional trial were 158.6, 156.3, 161.3 bpm and 46.7, 46.7 and 42.6 breaths⋅min^−1^ for the mindfulness, rest, and no-intervention, respectively, indicating athletes were engaged in high intensity exercise (34, 35).

### Relaxation interventions

**Figure 1:**
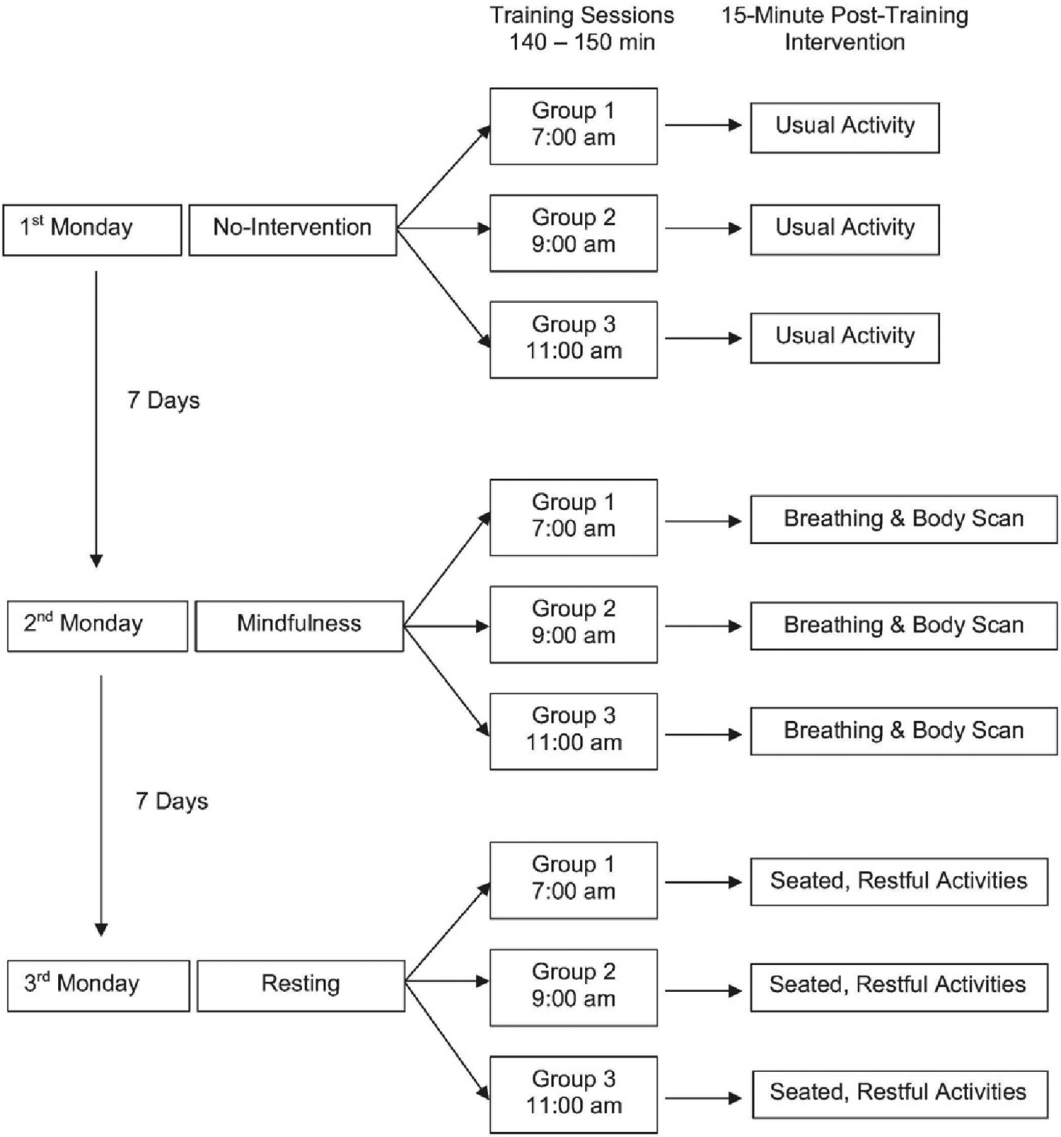
Schematic of the intervention implementation.

Each Monday, a different intervention was conducted immediately following an exercise training and lasted 15 min. On the 1st, 2nd and 3rd Mondays, the no-intervention, mindfulness, and resting interventions were administered, respectively. Each training day consisted of three training groups: 7:00 am, 9:00 am and 11:00 am. Each group consisted of a different set of athletes; however, the training exposure and post-training intervention were the same (see Figure 1). Athletes in the mindfulness group were instructed to lie supine on the floor in a darkened room and perform mindfulness exercises including breathing techniques and body scans. A professional trained in mindfulness guided the athletes throughout the session. For the rest group athletes were asked to remain seated in a lighted room and engage in restful activities such as rehydrating, refueling, conversing with teammates, etc. The no-intervention group was instructed to engage in their usual post-training activities (e.g., listening to music, showering, eating, standing around, horseplay).

### Parasympathetic reactivation

HRs, RRs and HRV were objectively measured pre-, during and post-intervention using armband monitors equipped with electrocardiographic and photoplethysmography (PPG) capabilities [Warfighter Monitor™ (WFM), Tiger Tech Solutions, Miami, FL]. The WFM has been previously validated in diverse populations (31–33, 36, 37). RR was calculated using an algorithm which used a low pass filter of the PPG signal. This removes the frequency components related to pulsatile flow and leaves only low frequency modulation related to respiration (38). HRV is defined as the time variation between heartbeats. The metrics used to evaluate HRV included the standard deviation of NN intervals (SDNN) and the root mean square of successive RR interval differences (rMSSD), described in detail elsewhere (39, 40). These metrics were calculated during a 5-min interval where the athletes were seated nearly motionless prior to the start of each training session. The anticipated decline of HRs and RRs and increases in HRV during and immediate post intervention represented parasympathetic reactivation, defined as the regained dominance of the PNS and withdrawal of the SNS following the cessation of exercise.

### Statistical analysis

Between-group differences in demographic characteristics were assessed via one-way ANOVA and Pearson's Chi-Square Test for continuous and categorical variables, respectively. Within- and between group differences in pre- and post-intervention HRs, RRs, rMSSD, and SDNN were assessed via repeated measures ANOVA and the familywise error consequent to multiple comparisons was accounted for using Tukey's Honest Significant Difference (HSD) test. All assumptions of the ANOVA were assessed and met. Means and standard deviations were estimated and the *a priori* threshold for statistical significance was set at *α* = 0.05. Statistical analyses were performed in MATLAB, version 2021b (MathWorks, Natick, MA). Importantly, an *a-priori* power analysis was not performed prior to study implementation. However, *post-hoc* power analyses were performed for detecting within- and between-group differences via repeated measures analysis of variance (ANOVA) model and showed that our study had 99.9% power to detect following differences for *mindfulness* vs. *no-intervention*: −46.1 bpm, −12.4 breaths⋅min^−1^, 14.4 ms for SDNN and 4.0 ms for rMSSD; *mindfulness* vs. *rest*: −18.0 bpm, −4.3 breaths⋅min^−1^, 5.7 ms for SDNN and 1.6 ms for rMSSD; *rest* vs. *no-intervention*: −28.0 bpm, −8.1 breaths⋅min^−1^, 8.4 ms for SDNN and 2.4 ms for rMSSD.

## Results

Table 2 presents the pre-intervention, post-intervention and change in HR, RR, rMSSD and SDNN across the three groups. At baseline (pre-intervention and post-exercise training), no statistically significant between-group differences in either HR, RR, or HRV metrics were reported. For HR, statistically significant reductions were observed for the *mindfulness*, *rest*, and *no-intervention* groups: −81.6, −66.4, and −40.9 bpm, respectively. Similarly, for RR, significant reductions were found for the *mindfulness*, *rest*, and *no-intervention* groups: −31.7, −26.9, and −19.0 breaths⋅min^−1^, respectively. Moreover, both the *mindfulness* and *rest* groups exhibited a larger reduction in both HR and RR compared to the *no-intervention* group, *p* < 0.0000. Additionally, the *mindfulness* group showed a larger reduction in HR and RR compared to the *rest* group, *p* < 0.05. For HRV, statistically significant increases in rMSSD and SDNN were observed for the *mindfulness, rest, and no-intervention* groups: + 8.6, + 7.3, + 4.8 ms and +27.4, + 22.7 and 14.4 ms, respectively. Like HR and RR responses, the *mindfulness* and *rest* groups exhibited larger increases in both HRV metrics compared to the *no-intervention* group, *p* < 0.0000. Also, the *mindfulness* group showed a larger increase in HRV compared to the *rest* group, *p* < 0.05.

**Table 2:** Within-Group differences in heart rate and respiratory rates, by group.

	Mindfulness		Rest		No intervention	
	Mean (SD)	% Difference	Mean	% Difference	Mean	% Difference
Pre-intervention						
Heart rate (beats⋅min^−1^)*	158.6 (7.6)	–	156.3 (27.5)	–	161.0 (7.5)	–
Respiratory rate (breaths⋅min^−1^)*	46.7 (3.9)	–	46.7 (8.7)	–	42.6 (4.3)	–
SDNN (ms)	19.6 (2.9)	–	20.2 (8.7)	–	18.7 (2.9)	–
rMSSD (ms)	9.3 (1.0)	–	9.4 (2.6)	–	9.0 (1.0)	–
Post-intervention						
Heart rate (beats⋅min^−1^)	77.0 (12.0)	–	89.9 (19.7)	–	120.1 (19.7)	–
Respiratory rate (breaths⋅min^−1^)	15.0 (4.5)	–	19.3 (6.7)	–	23.6 (7.5)	–
SDNN (ms)	46.9 (3.7)	–	42.9 (5.9)	–	33.1 (6.1)	–
rMSSD (ms)	17.9 (1.1)	–	16.7 (1.7)	–	13.8 (1.7)	–
Change**						
Heart rate (beats⋅min^−1^)	−81.6 (10.7)^a,b,c^	51.5	−66.4 (10.7)^a,b^	42.5	−40.9 (15.9)^a^	25.4
Respiratory rate (breaths⋅min^−1^)	−31.7 (7.4)^a,b,c^	67.9	−26.9 (7.4)^a,b^	40.4	−19.0 (7.2)^a^	44.6
SDNN (ms)	27.4 (3.4)^a,b,c^	58.4	22.7 (5.8)^a,b^	52.9	14.4 (5.27)^a^	43.5
rMSSD (ms)	8.6 (1.1)^a,b,c^	48.0	7.3 (1.8)^a,b^	43.7	4.8 (1.6)^a^	34.8

^a^ significant (*p* < 0.0000) within-group difference.

^b^ significant (*p* < 0.0000) Intervention vs No-Intervention.

^c^ significant (*p* < 0.05) Intervention vs Rest.

* no statistically significant differences in pre-intervention HR and RR were observed.

** change in HR and RR was calculated by the difference in post-intervention—pre-intervention to show the appropriate.

direction of the change.

**Figure 2:**
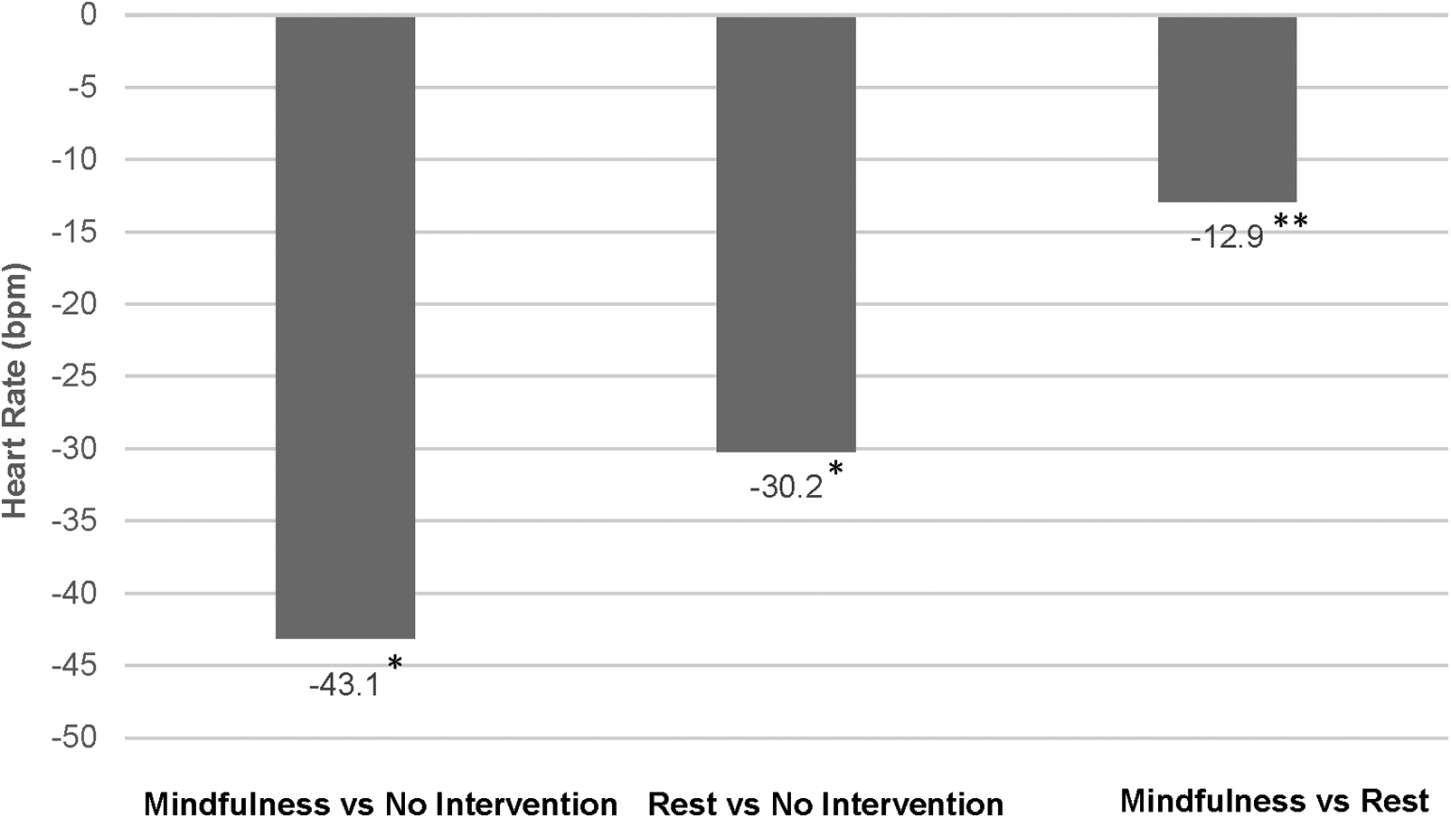
Differences in post-intervention heart rates between groups. *p < 0.0000; **p < 0.05.

**Figure 3:**
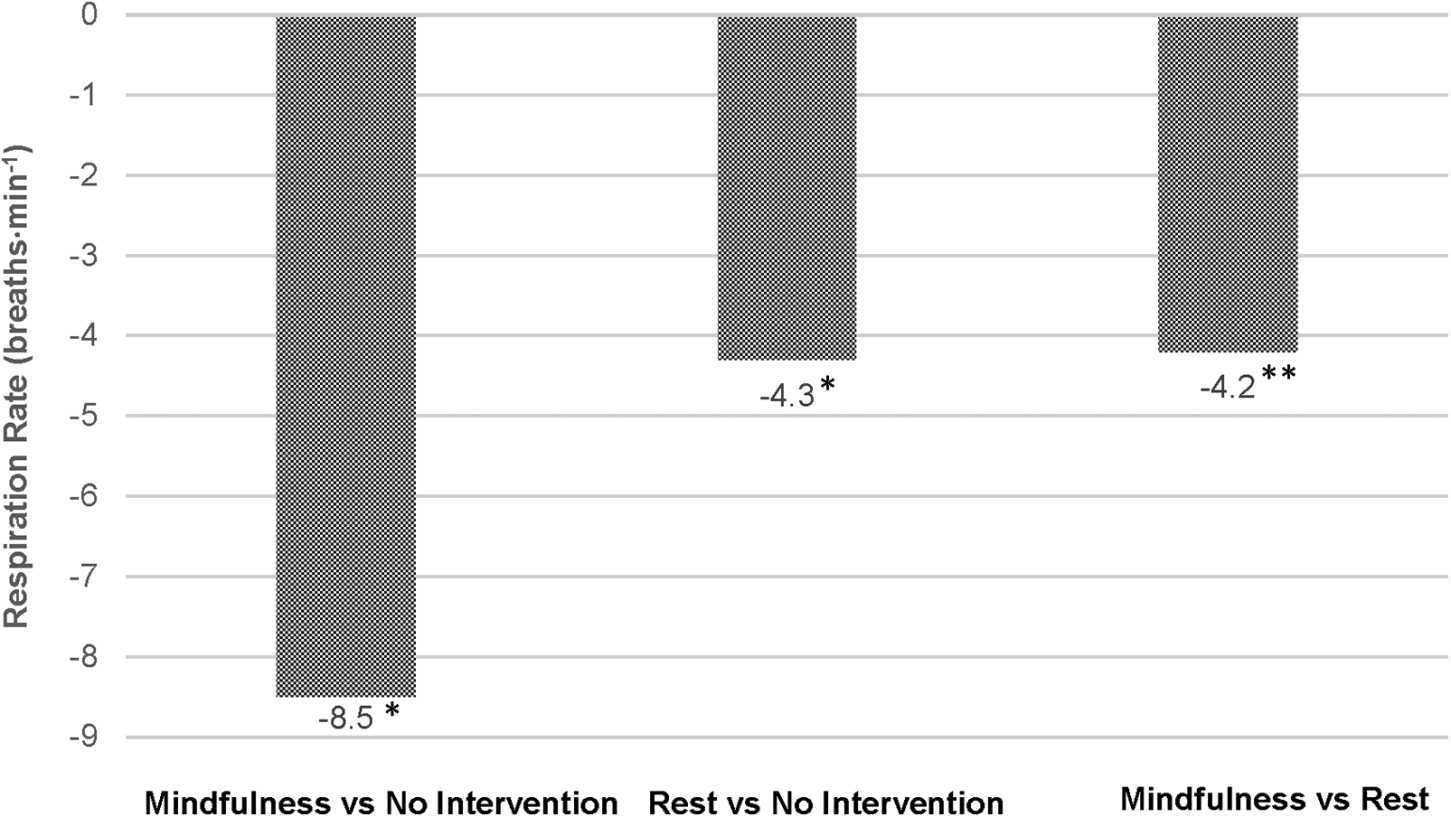
Differences in post-intervention respiration rates between groups. *p < 0.0000; **p < 0.05.

**Figure 4:**
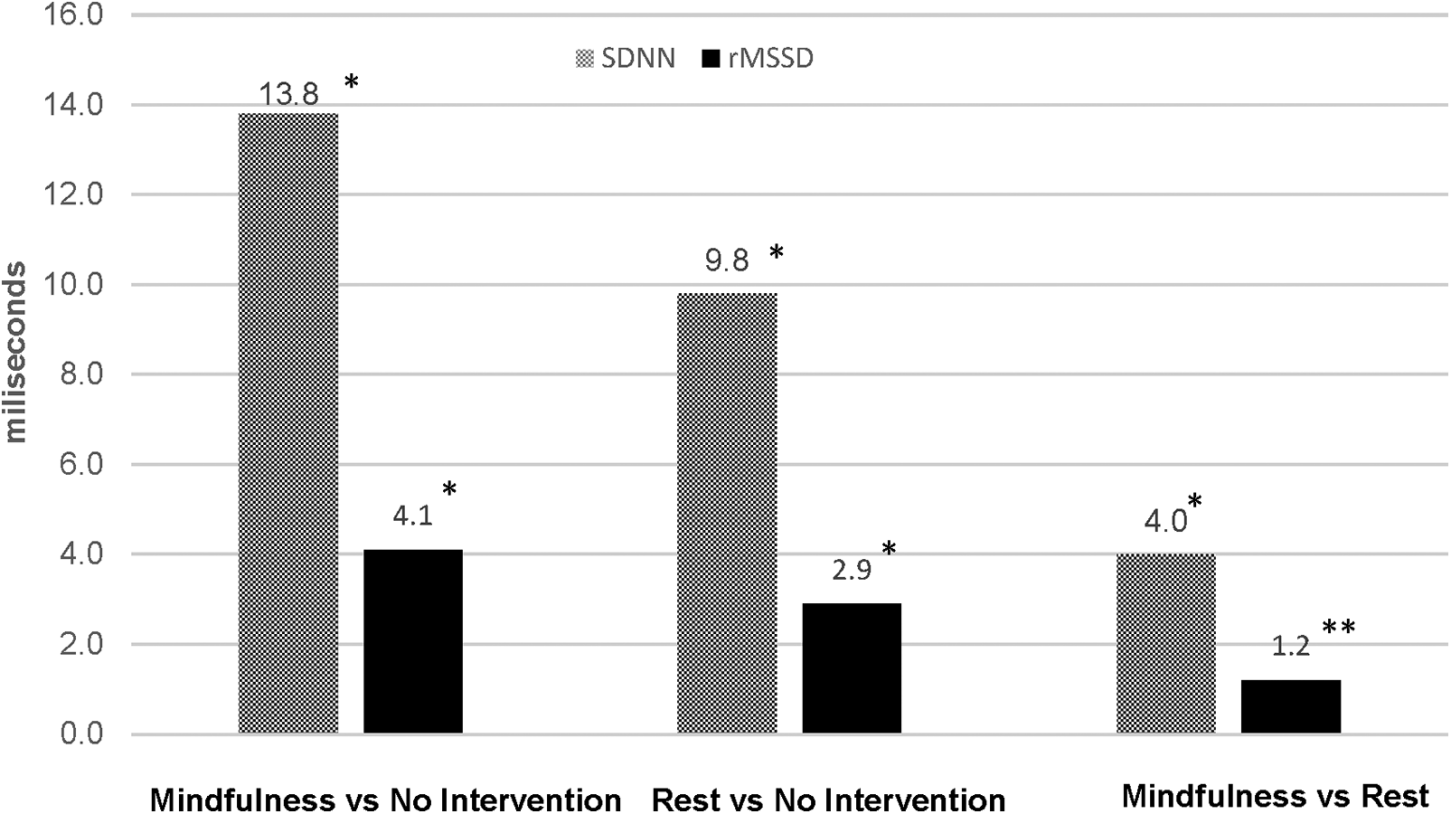
Differences in post-intervention heart rate variability between groups. *p < 0.0000; **p < 0.05.

Between-group differences in post-intervention HR, RR and HRV are illustrated in Figures 2–4, respectively. Both intervention groups elicited lower HR and RR post-intervention. Specifically, athletes in the *mindfulness* group exhibited a post-intervention HR, 43.1 bpm below the *no-intervention* group (77.0 vs. 120.1 bpm, respectively) and showed a significantly lower respiration rate following the intervention (15.0 vs. 23.6 breaths⋅min^−1^, respectively). Similarly, athletes in the *rest* group showed significantly lower post-intervention HR (−30.2 bpm, 89.9 vs. 120.1 bpm, respectively) and RR (−4.3 breaths⋅min^−1^, 19.3 vs. 23.6 breaths⋅min^−1^, respectively) compared athletes in the *no-intervention* group. Lastly, athletes using *mindfulness* following their training session elicited lower HR (−12.9 bpm, 77.0 vs. 89.9 bpm, respectively) and RRs (−4.2 breaths⋅min^−1^, 15.0 vs. 19.3 breaths⋅min^−1^, respectively) compared to athletes using the *rest* intervention. For HRV, athletes in the *mindfulness* group elicited significantly higher post-intervention rMSSD and SDNN values compared to the *rest* (+4.0 ms and +1.2 ms) and *no-intervention* (+13.8 ms and + 4.1 ms) groups. Additionally, athletes in the *rest* group exhibited significantly higher post-intervention rMSSD and SDNN values compared to the athletes in the *no-intervention* group (+9.8 ms and + 2.9 ms).

## Discussion

The purpose of this study was to evaluate the impact of two relaxation interventions on post-exercise training parasympathetic reactivation in a sample of Division-I, male football athletes. The main findings of this study were (1) both *mindfulness* and *rest* interventions elicited a greater parasympathetic reactivation compared to the *no-intervention* group, reflected by lower post-exercise HRs and RRs and higher HRV, (2) the two intervention groups showed the greatest within-group reduction in HRs and RRs and increases in HRV relative to the *no-intervention* group and, (3) at a smaller magnitude, the *mindfulness* group exhibited a stronger parasympathetic reactivation following exercise training compared to the *rest* group.

A novel aspect of this study was the observed parasympathetic reactivation during a 15-minute relaxation intervention that either included *mindfulness* or unguided *rest* following a routine training session. Specifically, athletes participating in *mindfulness* or *resting* practices, immediately after training, exhibited lower HRs and RRs and higher HRV compared to their *no-intervention* counterparts that resumed usual post-training activities. Importantly, at the start of the intervention, these athletes elicited post-exercise HRs associated with exercise performance at high intensity, which augments the increase in sympathetic activity, often requiring a longer period of recovery (5). Remarkably, following the *mindfulness* and *resting* interventions, the post-exercise HRs (77.0 ± 12.0 bpm and 89.9 ± 19.7 bpm, respectively) and RRs (15.0 vs. 19.3 breaths⋅min^−1^, respectively) exhibited were similar to values at rest and reached while performing activities of daily living (41, 42).Conversely, athletes in the *no-intervention* group, at the end of the 15-min trial, exhibited significantly higher HRs and RRs (120.1 ± 19.7 bpm and 23.6 ± 17.5 breaths⋅min^−1^, respectively) which are similar to values elicited during moderate intensity exercise (34, 35, 43). This finding contributes to the current literature as most studies focused on evaluating the effects of mindfulness practices on pre-competition psychological stressors like performance anxiety (22, 24, 44). Thus, the effects on physiological factors, like parasympathetic reactivation, were unclear. The observed reactivation of the PNS following *mindfulness* or *rest* post-training possibly suggests that athletes begin at least one aspect of their physiological recovery earlier. Consequently, these athletes may experience less fatigue during post-training activities, improved sleep, and readiness for subsequent training sessions.

Expectedly, all athletes elicited reductions in HR and RR and increases in HRV within 15 min following the cessation of training (45, 46). However, the magnitude of the reductions was markedly different amongst the groups, with the *mindfulness* and *rest* groups exhibiting the largest decreases in HR and RR compared to the *no-intervention* group. Specifically, the reductions in HRs and RRs among athletes in the *mindfulness* and *rest* groups were 200% and 160% and 168% and 140% larger relative to the *no-intervention* group. Similarly, the *mindfulness* and *resting* group exhibited higher increases for SDNN (190% and 160%) and rMSSD (179% and 150%), respectively, relative to the *no-intervention* group. These findings indicates that the *mindfulness* and *resting* techniques implemented post training were more effective in reactivating the PNS via lowering HR and RR and increasing HRV. This is further supported by larger proportion of recovery achieved among the *mindfulness* and *rest* groups relative to the *no-intervention* group. The post-intervention HR values were considerably closer to their respective baseline values for athletes in the *mindfulness* and *rest* groups, suggesting an accelerated recovery. Specifically, regarding HR, the *mindfulness* and *rest* groups were 78% and 53% recovered with the former 14 bpm above and latter above 28.6 bpm their baseline HRs. Comparatively, the *no-intervention* group appeared only 4.3% recovered with athletes, on average nearly 60 bpm above their baseline HR. Similar trends were shown for the HRV indices, however the proportion of recovery was considerably less: SDNN (40%–58%) and rMSSD (27%–37%), expectedly. Importantly, the extent of PNS reactivation was achieved in a small timeframe, only 15 min. The minimum time commitment required for effective PNS reactivation using these techniques may reduce the implementation burden making it a more efficacious and sustainable practice. Research shows that interventions of increased complexity demanding many resources and time, are prone to reduced compliance, minimized effects, and eventual withdrawal (47, 48).

Lastly, this study also observed significant differences in HR, RR and HRV between the two relaxation techniques. Specifically, athletes in the *mindfulness* group exhibited larger within-group reductions and lower post-intervention HRs and RRs (−81.6 vs. −66.4 bpm and −31.7 vs. −26.9 breaths⋅min^−1^, respectively) and higher SDNN and rMSSD values (+27.4 vs. +22.7 ms and +8.6 vs. +7.3, respectively) compared to the *rest* group. This finding is rather intuitive as the athletes in the resting group were exposed to more stimuli like lights, conversation, eating and drinking (49, 50). Moreover, the athletes were sitting upright resulting in increased skeletal muscle activation in addition to consuming foods yielding higher HRs, RRs and lower HRV. Despite the greater effectiveness of the *mindfulness* intervention, the *rest* group still elicited a significant effect on parasympathetic reactivation. This is encouraging as the resources (e.g., lighted room and seating) are minimal and specialized equipment is not required (e.g., mindfulness instructor or recording), lowering the burden of implementation (47, 48). Furthermore, providing a 15-minute resting period following training sessions may offer athletes an opportunity to bond with team members, positively influencing team cohesion (26).

### Strength and limitations

As with any study, there are strengths and limitations warranting attention. First, this study focused on evaluating the effects of two relaxation techniques following a routine football training session and found large effects on parasympathetic reactivation. To our knowledge, it is the first of its kind to (1) evaluate parasympathetic reactivation following acute training sessions using HR, RR and HRV metrics, (2) include a sample of collegiate, male football athletes and, (3) demonstrate large effects with objective measures and minimal participant burden (e.g., time, resources). These strengths may strongly influence the efficacy of these relaxation techniques. Some limitations of this study include lack of randomization. Although the athletes were not randomized to the three groups, no between-group differences were observed for demographic characteristics or pre-intervention measures and the athletes served as their own controls. This does not assume, however, the groups were equal, as unmeasured variables may have differed. Additionally, this study only evaluated the effects following one intervention trial. Thus, we cannot suppose that these findings, if repeated, would occur across several trials. Lastly, this study focused on one aspect of physiological recovery, which is a multifactorial process.

## Conclusions

The observations of the current study demonstrate that implementing brief relaxation techniques like guided mindfulness and low-key resting period following exercise training elicits a powerful effect on parasympathetic reactivation. This effect possibly facilitates an early onset of recovery, which is critical for athletes chronically exercising at high frequency, volume, and intensity, like collegiate football players. Importantly, this effect may reduce the physical, mental, and emotional demands occurring throughout the remainder of their day, which could also aid in a faster physiological recovery. Lastly, the brevity and low burden of this intervention increase its efficacy, especially as large effects were also observed for the resting group. As such, coaches may be more willing to implement and sustain these types of interventions. For future investigations, we strongly recommend researchers conduct longitudinal studies to evaluate longer-term effects on parasympathetic reactivation, sports performance, and other physiological recovery variables. Additionally, we suggest that forthcoming studies include a larger and more representative sample of collegiate athletes including diverse sports and females.

## Data Availability

The raw data supporting the conclusions of this article will be made available by the authors, without undue reservation.
